# Pharmacological Effects and Pharmacokinetic Profiles of Dehydroandrographolide

**DOI:** 10.1155/mi/4123997

**Published:** 2025-07-23

**Authors:** Chenggang Xu, Yu Shen, Lina Zhang, Feng Wang, Shuting Xiang

**Affiliations:** ^1^Department of Cardiology, XuanCheng City Central Hospital, Xuancheng, China; ^2^Department of Pharmacology, Anhui Medical University, Hefei, China; ^3^School of Pharmacy, Health Science Center, Xi'an Jiaotong University, Xi'an, China; ^4^Department of Pharmaceutics, China Pharmaceutical University, Nanjing, China

**Keywords:** anti-inflammatory mechanism, bioavailability, dehydroandrographolide, pharmacokinetics, pharmacological effects

## Abstract

Dehydroandrographolide (Deh) is one of the main active ingredients of the traditional Chinese medicine *Andrographis paniculata*. In recent years, its pharmacological research has made remarkable progress in the fields of antibacterial, anti-inflammatory, antitumor, and antiviral therapies. As a traditional herbal medicine, *Andrographis paniculata* has long been used in the clinical treatment of infectious diseases, immune disorders, and liver injury. Deh, the plant's core active molecule, has demonstrated therapeutic potential beyond that of its source, owing to its distinctive chemical structure and multitarget mechanism of action. Studies have shown that Deh not only inhibits inflammatory responses by regulating NF-κB, Nrf2, and other related pathways but also induces apoptosis and cycle blockade in tumor cells and exerts antiviral effects by interfering with the viral replication cycle. In this review, we systematically summarized the diverse pharmacological activities of Deh and its molecular mechanisms, drawing attention to its potential role in the treatment of inflammation-related diseases. We hope this work will serve as a theoretical reference for designing innovative drugs based on natural products and encourage the clinical translation of *Andrographis paniculata*'s active ingredients.

## 1. Introduction

*Andrographis paniculata*, an annual herb belonging to the genus *Andrographis* in the family *Acanthaceae*, is traditionally used in Chinese medicine. The dried aerial parts of the plant are valued for their ability including clearing heat, detoxifying, cooling blood, and reduce swelling. Native to Southeast Asia, *Andrographis paniculata* is often referred to as a “natural antibiotic” because of its impressive medicinal properties. It is also commonly used as a dietary supplement in Europe. In recent years, it has been extensively cultivated in southern China, particularly in Guangdong and Guangxi provinces, becoming an essential part of the rich heritage of traditional Chinese medicine. Modern research has revealed that the medicinal parts of *Andrographis paniculata* contain a wide array of active compounds, including diterpene lactones, flavonoids, and phenylpropanoids [1, 2]. Among them, the diterpene lactone dehydroandrographolide (Deh) is one of the primary bioactive ingredients in *Andrographis paniculata*. The 2020 edition of the *Chinese Pharmacopoeia* explicitly lists four diterpene lactones, including andrographolide and Deh, as quality control markers for *Andrographis paniculata*, emphasizing the critical role of Deh. Currently, preparations of Deh derivatives such as Deh succinate (DAS) injection and Potassium Deh succinate injection (PDSI), have been widely used in clinical settings, primarily for the treatment of various infectious diseases [3].

With the advancement of modern pharmacological research, the diverse therapeutic effects of Deh have gained increasing recognition. Studies have demonstrated that Deh possesses significant antibacterial, anti-inflammatory [4, 5] antitumor, and antiviral properties, while also effectively enhancing immune function [6, 7]. Particularly in the field of anti-inflammation research, Deh has shown the potential to surpass other *Andrographis paniculata* lactones. Notably, Deh not only exhibits stronger pharmacological activity but also offers a better safety profile compared to andrographolide [8, 9]. However, most current research on *Andrographis paniculata* has primarily focused on andrographolide, while systematic reviews on Deh remain scarce. This gap limits a comprehensive understanding of the therapeutic effects and underlying mechanisms of the monomeric components of *Andrographis paniculata*. Given Deh's notable anti-inflammatory properties and its potential for clinical applications, this review provides a systematic overview of the latest research on the pharmacological activities of Deh and its mechanisms of action, building on previous literature. The aim is to establish a scientific foundation for further development and clinical use of Deh.

## 2. Pharmacological Action and Mechanism of Deh

**Figure 1:**
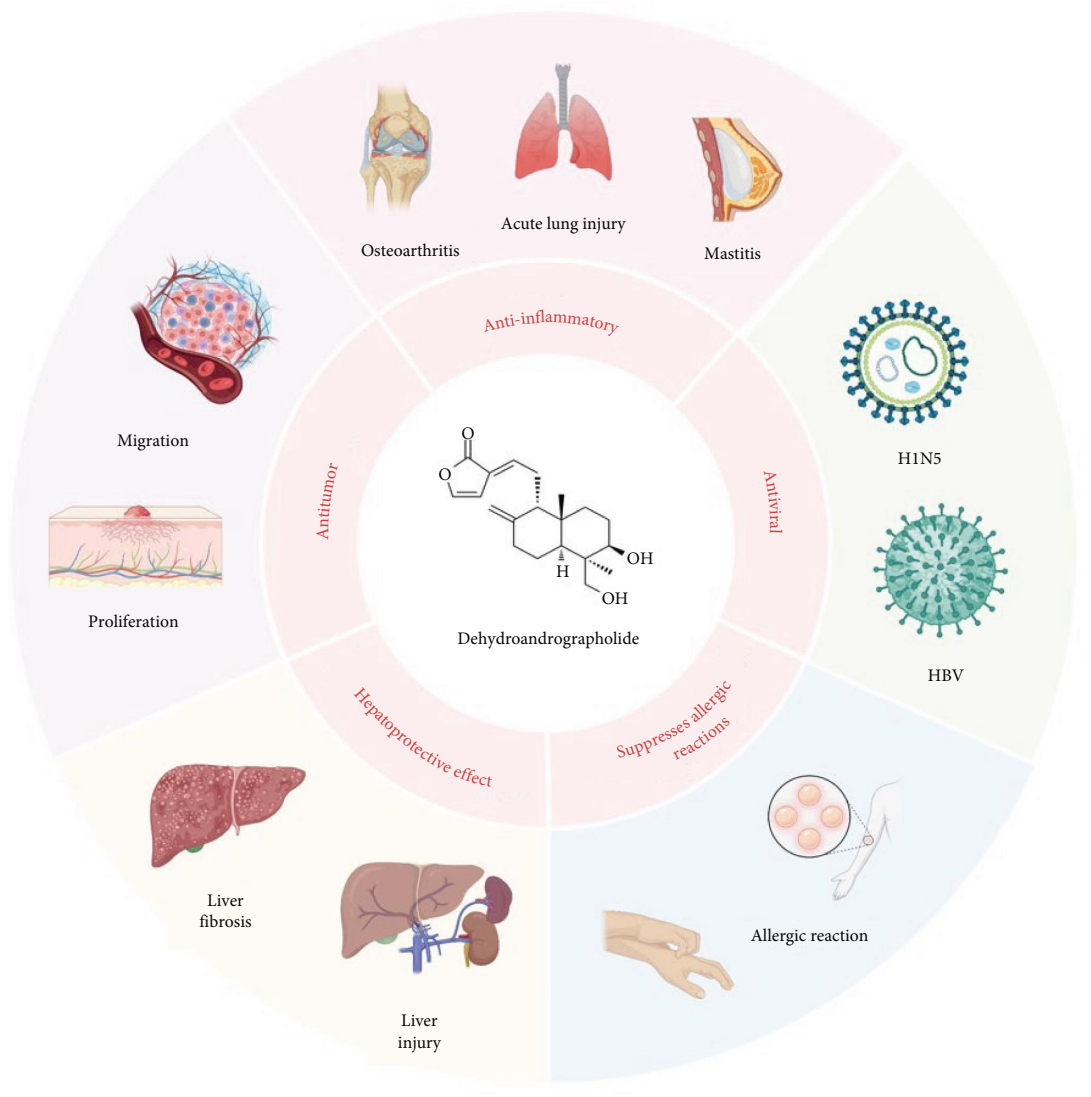
The main pharmacological activities of Deh.

Deh (C_20_H_28_O_4_) is a diterpene lactone, as shown in Figure 1, and its hydrochloride salt is the primary chemical form used for medicinal purposes. The most representative examples are DAS Injection and PDSI, both of which have been approved in China for the treatment of viral pneumonia and upper respiratory tract infections. Their clinical efficacy has been well established, with PDSI in particular showing significant benefits in pediatric infectious diseases such as pneumonia and epidemic parotitis. For example, PDSI has demonstrated notable clinical efficacy in the treatment of pediatric infectious diseases. In a meta-analysis of pediatric pneumonia, nine randomized controlled trials involving 1056 children showed that PDSI, when combined with conventional therapy, significantly improved the overall response rate and accelerated the resolution of fever and cough symptoms [10]. Similarly, in the treatment of pediatric epidemic parotitis, a pooled analysis of 11 trials comprising 818 pediatric patients indicated that the combination therapy group achieved superior outcomes in terms of overall efficacy, fever reduction, and parotid gland swelling resolution, while also significantly lowering the incidence of complications compared to the control group [3]. In addition to its use in treating infectious diseases, the diverse pharmacological effects of Deh have been demonstrated in numerous studies [11–13]. These effects are categorized according to their pharmacological actions, as shown in Figure 1 and Table 1.

### 2.1. Antiviral Effect

**Figure 2:**
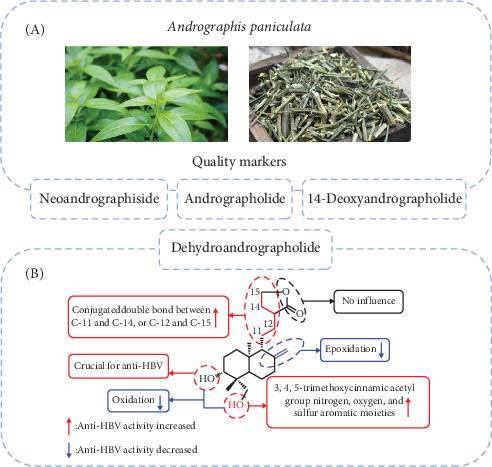
Dehydroandrographolide is recognized as a quality marker of andrographis paniculata and possesses antiviral properties. (A) Dehydroandrographolide is one of the quality markers of andrographis paniculata. (B) Structure activity relationships of dehydroandrographolide and andrographolide derivatives anti-HBV activity [38]. Copyright 2014 Elsevier Ltd.

Natural products serve as excellent starting points for the design and development of new drug-like candidates [34, 35]. Compounds derived from the traditional Chinese medicine *Andrographis paniculata* have demonstrated efficacy against various viruses, including hepatitis B virus (HBV), influenza A (H1N5) virus [29], and the Zika virus [31, 36]. Among these, Deh is considered a major contributor to *Andrographis paniculata*'s therapeutic properties [37]. Both Deh and andrographolide exhibit inhibitory activity against HBV DNA replication, with IC_50_ values of 22.58 and 54.07 μM, respectively. The selectivity index (SI) values for Deh and andrographolide were 8.7 and 3.7, respectively, indicating their potential for further development as antiviral agents. Chen et al. [38] synthesized 48 derivatives of Deh and andrographolide to evaluate their anti-HBV properties (Figure 2). The study revealed three key findings: (1) compounds with a free hydroxyl group at the C-3 position of 19-O-substituted derivatives exhibit higher anti-HBV activity compared to other analogs; (2) the methylene group at C-15 is essential for anti-HBV activity; and (3) both the bond between C-8 and C-17 and the conjugated double bonds between C-11 and C-14 or between C-12 and C-15 are crucial for maintaining anti-HBV activity.

hBD-1 is a key antimicrobial peptide in human epithelial defense against infections [30]. It can reduce viral infectivity by binding to the virus surface and disrupting the binding process between the virus and host cells, thereby preventing the virus from attaching to host cell receptors. hBD-1 is expressed in various epithelial tissues, including the prostate, kidneys, and the tubular lumen of the genitourinary tract. As early as 2010, Shao et al. [28] discovered that Deh upregulated the expression of hBD-1 mRNA and protein in human mammary epithelial cells, with an optimal concentration of 80 μM and maximum expression achieved after 8 h. In addition, the Deh derivative, 14-Deoxy-11,12-dehydroandrographolide (DAP), strongly inhibited H5N1 virus replication by reducing viral nucleoprotein production and significantly decreasing the expression of inflammatory cytokines (TNF-α, IL-6, IL-8, IFN-α, IL-1β, and IFN-β) stimulated by H5N1 infection.

It is important to note that viral infections and inflammatory responses are closely intertwined. While inflammation in a vital defense against viral infections, excessive or uncontrolled inflammation can damage host tissues. Deh, as a natural antiviral agent, has shown great promise in antiviral therapy through mechanisms such as inhibiting viral replication, modulating immune responses, and reducing inflammation. However, more research is needed to thoroughly evaluate the antiviral activities of Deh and its derivatives against various viruses, as well as to explore their pharmacokinetic (PK) properties and long-term safety. Additionally, because Deh contains multiple unstable chemical sites, which significantly limit its bioavailability, structural modifications, the development of multiple derivatives, and strategies to enhance its chemical stability and bioactivity remain critical areas for future investigation.

### 2.2. Anti-Inflammatory Effect and Mechanism

Inflammation is an adaptive response triggered by external harmful stimuli (pathogens, toxins, or injuries). and is regulated by a network of inflammatory mediators. Key signaling pathways, including NF-κB, Akt/Nrf2, and AMPK, are pivotal in driving the initiation and progression of various inflammatory diseases, such as acute lung injury (ALI), liver injury, arthritis, and mastitis, by modulating the secretion of inflammatory factors. Ling et al. [39] conducted in silico molecular docking simulations, demonstrating that Deh exhibits a strong affinity for several anti-inflammatory targets. In fact, Deh showed greater interaction with target receptors than classical anti-inflammatory agents like diclofenac and fenoprofen [39]. Furthermore, Deh significantly inhibited LPS-induced production of TNF-α and GM-CSF, achieving half- maximal inhibitory concentrations at 2.4 and 8.9 µM, respectively, comparable to dexamethasone levels of efficacy.

**Figure 3:**
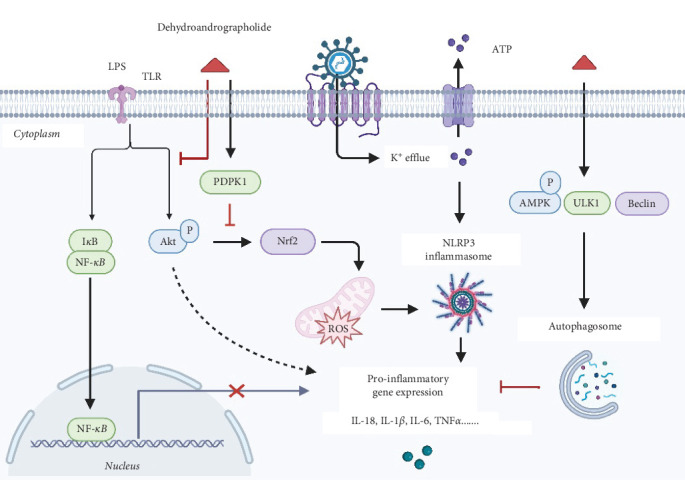
Anti-inflammatory mechanism of Deh.

ALI often arises from pathogens such as bacteria and viruses. Excessive production of inflammatory mediators in lung tissues initiates a systemic inflammatory response, exacerbating the degree of lung damage and making inflammation control crucial for the effective management of ALI [40, 41]. Pu et al. [17] demonstrated in both in vivo and in vitro ALI models that Deh significantly reduced inflammation and oxidative stress by inhibiting NLRP3-mediated pyroptosis (Figure 3). Additionally, Deh was shown to suppress the Akt/Nrf2 pathway, reducing reactive oxygen species (ROS) production and mitigating mitochondrial damage [17]. The analogous biological activities of Deh and its parent compound, andrographolide, can be attributed to their structural similarities. Specifically, the conjugation of double bonds at the C-12 and C-13 positions to the lactone ring forms a key active center. This structural feature is crucial for Deh's interaction with inflammation-related targets, such as NF-κB and NLRP3. For example, Lu et al. [14] observed that Deh could inhibit NF-κB activation, resulting in suppressed production of pro-inflammatory cytokines like IL-6 and TNF-α. They proposed that α7nAchR might be a potential target for Deh's anti-inflammatory effects [14]. These findings provide a solid foundation for considering Deh as a therapeutic option in ALI treatment. However, despite its potent anti-inflammatory properties, Deh's poor solubility and low bioavailability limit its clinical use. To overcome this limitation, researchers have developed a Deh–phospholipid complex [42], which demonstrated excellent aerodynamic properties (with a fine particle fraction of 60% and an aerodynamic diameter of 2.3 μm). This complex allowed efficient deposition in the alveolar region. In a mouse model of ALI, the complex significantly enhanced Deh retention in the respiratory epithelium following lung delivery, extended the duration of its anti-inflammatory effects and notably improved its bioavailability and tissue targeting [4].

Cholestatic liver injury is often characterized by inflammatory cell infiltration and elevated levels of inflammatory factors in both liver tissue and plasma. These factors reduce bile flow, further exacerbating cholestasis. Weng et al. [15] demonstrated in a mouse model of cholestatic liver injury induced by LPS administration into the ileocecal vein that treatment with 50 mg/kg of Deh significantly reduced the release of inflammatory factors and inflammatory cell infiltration in both liver tissues and plasma. This effect was attributed to Deh's inhibition of LPS binding to hepatic Kupffer cells, preventing the activation of NF-κB and mitigating the LPS-induced inflammatory response. Furthermore, in an LPS-induced mouse mastitis model, Deh significantly inhibited the expression of IL-6, IL-1β, and TNF-α in EpH4-Ev mouse mammary epithelial cell line [16]. Notably, the anti-inflammatory effect of Deh was blocked when an AMPK inhibitor was introduced, suggesting that its mechanism of action involves activating the AMPK/Beclin/ULK1 signaling pathway to promote autophagy. Kong et al. [18] found that oral administration of Deh (2 mg / kg /day) to collagen-induced arthritis (CIA) rats alleviated synovitis and cartilage damage by inhibiting the release and chemotaxis of cytokines from LPS/TNF-α-activated neutrophils. The specific mechanism was identified as Deh's ability to inhibit neutrophil activation through binding to LMIR3, which in turn alleviated CIA. A placebo-controlled clinical trial assessing the effects of a Deh-containing combination on 18 patients with rheumatoid arthritis demonstrated that this combination significantly alleviated clinical symptoms, including pain and inflammation [43].

These studies suggest that Deh holds therapeutic potential for treating inflammatory diseases such as rheumatoid arthritis, ALI, and liver injury, given the crucial role inflammation plays in their onset and progression. The anti-inflammatory effects of Deh are not mediated through a single pathway but rather involve a complex, multipathway mechanism. It primarily exerts its effects by inhibiting the release of inflammatory mediators, modulating immune cell function, and blocking key inflammatory signaling pathways [44], as confirmed in various in vitro and in vivo models. However, when exploring the broader pharmacological effects of Deh in inflammation-related diseases, it remains important to focus on the shared mechanisms underlying these conditions and to clarify the specific therapeutic scope of Deh. For example, autophagy and inflammation are closely interconnected, working together to maintain the body's immune homeostasis. It has also been shown that Deh exerts a cardioprotective effect by promoting the completion of autophagy in DOX-induced cardiotoxicity (DIC) [45]. Whether Deh holds potential therapeutic value in other inflammatory-related diseases where autophagic processes play a key role warrants further investigation.

### 2.3. Antitumor Effect and Mechanism

Recent studies have shown that Deh not only possesses significant anti-inflammatory effects but also demonstrates notable antitumor activity across various tumor models. Deh inhibits tumor cell proliferation and metastasis while also inducing apoptosis [23]. One study revealed that Deh inhibited SW620 colon tumor cell proliferation in a dose and time-dependent manner and also reduced their migration and invasion capacities [19]. Similarly, Liu et al. [21] found that Deh inhibited the proliferation of osteosarcoma cells in a dose-dependent manner, leading to cell cycle arrest and further inhibiting tumor growth. Specifically, Deh not only suppressed tumor cell proliferation but also reversed the epithelial-mesenchymal transition (EMT) of osteosarcoma cells by targeting SATB2, thereby reducing their metastatic potential. This inhibitory effect on EMT was also observed in SCC9 oral tumor cells [20]. Hsieh et al. [22] demonstrated that Deh induces autophagy in oral tumor cells by regulating p53 expression, activating JNK1/2, and inhibiting Akt and p38 signaling pathways. Additionally, Deh was shown to be effective in inhibiting tumor formation in an established oral tumor xenograft model. Lei et al. [24] further revealed that Deh not only inhibited the proliferation of endometrial tumor cells but also induced apoptosis. The underlying mechanism appears to involve the upregulation of Caspase-3 expression and the downregulation of Bcl-2 levels [24]. In recent years, researchers have focused on studying the structural relationship of Deh derivatives to identify compounds with enhanced antitumor activity. It is believed that the lactone ring and 1,3-diol structure of Deh are critical for its ability to inhibit tumor cell growth [46]. By modifying the 3- and 19-positions of Deh and introducing a cyclic phosphonate structure, new derivatives were synthesized that exhibited lower fat solubility, reduced toxicity, and enhanced antitumor activity compared to Deh itself [47, 48].

Overall, Deh, as a natural antitumor compound, primarily exerts its effects through multiple mechanisms, including the inhibition of tumor cell proliferation, induction of autophagy, blockade of tumor cell cycle, and suppression of tumor cell migration and invasion. However, the antitumor action of Deh is not solely limited to directly killing tumor cells; it also involves mechanisms that improve the tumor microenvironment and modulate the immune response, which could offer new perspectives for its application in cancer therapy. For instance, studies have shown that andrographolide inhibits macrophage and lymphocyte proliferation at high concentrations, while exhibiting immunostimulatory activity at low concentrations [49]. Given that Deh shares a closely similar chemical structure to andrographolide, it is worth further investigating whether Deh exhibits similar immunomodulatory effects.

### 2.4. Suppression of Allergic Reactions

An allergic reaction is an exaggerated inflammatory response that involves processes such as immune cell activation, release of inflammatory mediators, and tissue damage. When an allergen re-exposure occurs, the antigen binds to IgE on sensitized cells, triggering mast cell (MC) degranulation and the release of various inflammatory mediators, including histamine, prostaglandins, and leukotrienes. These mediators cause vasodilation and increased vascular permeability, leading to typical allergic symptoms, including skin redness, swelling, itching, and respiratory distress.

Deh can inhibit IgE-mediated allergic reactions and reduce foot and plantar swelling in mice by suppressing MC degranulation [4, 32]. It dose-dependently decreases serum levels of histamine, TNF-α, MCP-1, IL-13, and IL-4, further inhibiting IgE-mediated allergic responses. Mas-related G protein-coupled receptor X2 (MRGPRX2) mediates MC activation, which is a key target for the treatment of allergic diseases. Che et al. [33] found that Deh negatively regulates mrgprx2-mediated MCs activation through leukocyte mono-immunoglobulin-like receptor 3 (LMIR3, also known as CD300f), effectively inhibiting pseudo allergic responses. Specifically, CD300f acts as a negative regulator of MC activation. Deh also upregulates the phosphorylation levels of Src homology region 2 domain-containing phosphatase (SHP)-1 and SHP-2, which are key kinases involved in the negatively regulated signaling pathway associated with CD300f. By modulating this pathway, Deh inhibits allergic responses such as chronic urticaria and atopic dermatitis.

### 2.5. Hepatoprotective Effect

Liver injury and fibrosis are typically associated with a pronounced inflammatory response, characterized by elevated secretion of pro-inflammatory cytokines and chemokines, along with the infiltration of macrophages, lymphocytes, and neutrophils. This inflammation substantially contributes the progression of fibrosis and can also impact liver function recovery and the overall progression of the disease. As a result, anti-inflammatory therapy is a critical strategy to mitigate liver injury and prevent further worsening of fibrosis. Jing and Li [26] administered Deh at a dose of 100 mg/kg to mice in a carbon tetrachloride-induced hepatic fibrosis model. They found that Deh significantly increased the superoxide dismutase (SOD) levels in liver tissues and improved the degree of inflammatory cell infiltration and collagen deposition in the liver [26]. Compared to the model group, the treated group showed significant reductions in serum levels of alanine aminotransferase (ALT), aspartate aminotransferase (AST) activities, and malondialdehyde (MDA) levels in liver tissue. The mechanism underlying these effects was linked to a reduction in oxidative stress, inhibition of hepatocyte apoptosis, and decreased activation of hepatic stellate cells (HSCs). Acetaminophen-induced acute liver injury is a serious health condition that often presents with abnormally high levels of AST and ALT, along with inflammation. Similarly, in a mouse model of drug-induced acute liver injury [27], Deh was effective in alleviating liver damage by significantly increasing glutathione and serum SOD levels. It also reduced serum levels of AST and ALT, with mechanisms related to the activation of NRF2, promotion of energy metabolism, and induction of autophagy.

Cholestasis, a condition characterized by impaired bile flow or excretion, is another major contributor to liver injury. Consequently, the accumulation of bile components, particularly bile acids, within the liver can lead to hepatocyte damage [50]. Weng et al. [25] confirmed the hepatoprotective effects of Deh using a mouse model of bile duct ligation (BDL) and human LX-2 cells. The results demonstrated that Deh treatment significantly reduced hepatic injury and fibrosis formation after BDL in mice [25]. Moreover, Deh enhanced hepatic adaptive responses, inhibited HSC activation, and reduced extracellular matrix (ECM) deposition, which may contribute to the reduction of hepatic fibrosis in cholestatic diseases.

In short, Deh exerts therapeutic effects in liver injury and other inflammatory diseases through multiple mechanisms, including anti-inflammatory, antioxidant, and immune-modulatory actions. By attenuating liver inflammation, promoting cellular repair, and reducing fibrogenesis, Deh presents a promising therapeutic option for these conditions. Its multifaceted effects highlight its potential as a treatment for various inflammation-related diseases.

## 3. PK Study of Deh

Several PK studies of Deh have been conducted in various species, including rats, Beagles, and chickens [51, 52]. The main PK parameters from these studies are summarized in Table 2. The results are summarized based on absorption, distribution, metabolism, and excretion (ADME), while also considering disease-related influences on these parameters. Due to its low water solubility, Deh poses challenges for oral administration in animal experiments. Due to its low solubility, special solvents or suspensions are often required. As a result, the choice of solvents by different researchers has influenced the absorption process to some degree. Additionally, variations in solvent selection, formulation preparation, and the final Deh content [59, 60] have led to inconsistencies in PK parameters across studies. Nevertheless, the general PK profiles remain broadly consistent.

After oral administration of 120 mg/kg of Deh to healthy SD rats, the mean maximum plasma concentration was 4.19 ± 1.76 μg/mL, and Deh could be detected in the plasma within 1 h of administration. Deh exhibits rapid absorption and metabolic elimination, with an oral bioavailability of 11.92% [56]. Based on these findings, oral administration may not be the optimal route for effective Deh delivery. Liu et al. [54] administered a single dose of 5 g/kg of *Andrographis paniculata* ultramarginata to chickens, based on their body mass. The study found that the metabolic process of Deh (*t*_1/2_ = 143.0 ± 12.8 min, *t*_1/2Ka_ = 9.77 ± 1.22 min) was significantly different from andrographolide (*t*_1/2_ = 104.8 ± 12.8 min, *t*_1/2Ka_ = 23.2 ± 2.6 min) [54]. Deh exhibited faster absorption and a longer in vivo retention time, which may help explain its stronger pharmacological effects compared to andrographolide. Similarly, in a clinical trial with eight healthy volunteers, *Chuanxinlian* tablets containing Deh showed a slower absorption rate in humans, with peak concentration occurring ~1.5 h after oral administration [53].

Cunfang et al. [55] observed a bimodal pattern in the blood concentration–time curve of Deh after oral administration of *Chuanxinlian* tablet suspension to rats. This phenomenon may be attributed to hepatic-intestinal or gastrointestinal circulation, as well as the presence of dual absorption sites. Nevertheless, the exact mechanism behind this effect remains to be elucidated [55]. The absorption and metabolism of Deh in the intestine were found to be stable. In a rat intestinal perfusion model, the absorption rate constant (Ka) of Deh slightly decreased as the pH increased from 5.34 to 7.40, with the ileum identified as the optimal absorption site for Deh [61]. Notably, the transit time of Deh in the colon was approximately eight times longer than in the small intestine, and the absorption in the colon even surpassed that in the small intestine. Given these intestinal absorption characteristics, Deh could be structurally modified to create a locally released drug for targeted therapeutic applications.

Plasma protein binding significantly influences a drug's distribution and biological effects within the body. Deh has been found to exhibit moderate degree of such binding. Wu and Zang [62] employed ultrafiltration and high-performance liquid chromatography (HPLC) to assess the protein binding of Deh in BSA, human plasma, and rat plasma. The protein binding of Deh to these substances was found to be 71.50% ± 1.50% for BSA, 79.91% ± 2.51% for human plasma, and 82.41% ± 2.05% for rat plasma [62, 63]. Following tail vein injection of Deh derivatives in rats, the drug was distributed across various tissues, including the heart, liver, spleen, lungs, kidneys, and intestines. The drug concentration, ranked from highest to lowest, was observed to be kidney > colon > liver > lung > heart > spleen > stomach [64]. In a study by Wei-Ya et al. [65], DAS was administered via both intratracheal and intravenous routes. The results indicated that the absolute bioavailability of DAS after intratracheal administration was 47.3%, and relative to intravenous administration, it increased the drug concentration in rat lung tissues by over 80-fold. This suggests that inhalation administration of DAS is a convenient and effective alternative to intravenous delivery.

Despite sharing similar structures and biological activities, Deh and andrographolide exhibit markedly different PK profiles and intestinal absorption mechanisms. For example, andrographolide has poor oral bioavailability (2.67%) due to its rapid conversion and efflux via P-glycoprotein (P-gp). In contrast, Deh is not affected by efflux transporters like P-gp or Breast Cancer Resistance Protein (BCRP) in its translocation. In fact, Deh exhibits a significant inhibitory effect on P-gp in Caco-2 cells (IC50 = 77.80 μM) [66]. Kemin et al. [67] investigated the excretion of Deh derivatives in bile, urine, and feces after intramuscular injection in rats. At 24 h postadministration, 24.9% of the total dose was excreted via urine, 14.9% via bile, and 13.9% via feces. Only trace amounts of the drug remained in the system after this period. Additionally, Bai et al. [68] identified 35 metabolites in urine, bile, plasma, and fecal samples from rats given Deh at a dose of 95 mg/kg. Of these metabolites, 33 were further confirmed by stable isotope labeling. The primary metabolic pathways of Deh include hydroxylation, sulfonation, sulfate conjugation, and glucuronidation [68, 69].

It is important to note that Deh exhibits significant differences in PK parameters under pathological versus normal conditions. For instance, in a rat model of cholestasis, the *T*_max_ and *C*_max_ values of Deh nearly doubled [52], indicating slower absorption, which may be attributed to gastrointestinal dysfunction associated with liver injury [70]. In a rat model of ulcerative colitis, Deh showed a similar PK profile [57], with the AUC_0–∞_ increasing from 2355.73 to 4794.15 ng h/mL. A separate study reported no change in plasma concentrations of Deh following administration of 20 mg/kg in mice with ALI [58]. However, compared to normal mice, Deh exhibited higher maximal concentrations, larger AUCs, and longer elimination half-lives. These findings suggest that Deh may alleviate lung edema and inflammation in ALI mice, implying that lung injury may alter the PK properties of Deh, resulting in enhanced pulmonary distribution and retention. Therefore, further investigation of the PK profile of Deh under pathological conditions is warranted.

It is essential to note that research on the in vivo distribution of Deh still requires further exploration. Currently, the mechanisms underlying Deh's accumulation, metabolism, and clearance across various tissues and organs remain unclear, and its target distribution and PK properties require systematic investigation. Future studies should focus on understanding the distribution patterns, tissue selectivity, and potential accumulation of Deh in different tissues. This will help clarify its efficacy and safety across different disease states, optimize its administration protocols, and enhance its clinical applicability. Moreover, it will provide a scientific foundation for the precise treatment of Deh in other inflammation-related conditions.

## 4. Conclusion and Perspectives

Deh shows significant promise for future research and application due to its wide range of pharmacological effects, particularly in anti-inflammatory, antitumor, and immunomodulatory areas [45, 71]. This review summarizes recent key findings, which can serve as a valuable reference for further in-depth studies. Although numerous studies have examined the pharmacological effects of Deh, its mechanisms of action remain to be fully elucidated. Most studies focus on pharmacodynamics (PDs), specific signaling pathways, or individual targets, without fully addressing the interconnections between these pathways and targets from a holistic perspective. For instance, Deh's potential therapeutic effects on inflammatory diseases indicate possible connections between various disease-related targets. Therefore, future research on Deh may leverage network pharmacology and multiomics technologies to construct a “component-target-disease” interaction network, thereby enabling a comprehensive analysis of its specific targets and signaling pathways. This approach will help to unravel the mechanisms underlying its synergistic, multitarget actions [1, 9].

Deh derivative injections, such as Xiyanping, Yanhuning, Chuanhuning, and Lianbizhi, are traditional Chinese medicine products currently used in clinical settings to treat various inflammatory conditions, including upper respiratory tract infections, bacillary dysentery, and pediatric pneumonia [72]. However, despite their efficacy, these drugs are associated with some adverse effects. As a result, researchers are exploring more advanced delivery systems to minimize the incidence of these side effects and improve patient outcomes [73–75]. In the future, technologies such as nanocarriers, liposomes, and microemulsions could be employed to enhance the bioavailability of Deh, thereby improving its therapeutic efficacy in the body. The storage of *Andrographis paniculata* and its pharmaceutical processing are influenced by factors such as temperature and pH, with prolonged storage leading to an increase in the content of Deh [76]. In vivo, the environment is much more complex. Further investigation is needed to determine whether and how Deh undergoes metabolic transformation in vivo. Given the complexity of multilevel physiological processes in biological systems, traditional research methods often fall short of providing in-depth insights at the mechanistic level. PKs/PDs research on active ingredients derived from natural plants is essential for advancing their clinical translation and optimizing drug use [77]. One promising approach is the use of mathematical models based on quantitative systems pharmacology (QSP) that can reveal disease progression from a dynamic perspective. These models predict PD responses and potential adverse effects of drug interventions by integrating multiphysiological disease mechanisms and drug–target interactions. Additionally, QSP can systematically combine preclinical multiomics data with clinical observations. This modeling technique has demonstrated success in oncology, cardiovascular, and metabolic disease research [78, 79]. Applying QSP to Deh's ADME analysis could facilitate systematic extrapolation of preclinical data to human PK parameters, thereby providing a strong theoretical and experimental basis for Deh's clinical application in inflammation-related diseases.

In general, research into the pharmacological mechanisms of Deh is progressing steadily, particularly in the areas of anti-infective and anti-inflammatory effects. Clinical trial results indicate that DAS is rapidly distributed following administration, cleared quickly from the body, exhibits nonlinear PK behavior, and demonstrates good overall tolerability [3, 80]. While DAS shows favorable safety and notable anti-inflammatory potential, its short half-life and low urinary excretion of the parent compound suggest a complex metabolic process in vivo, with its metabolites likely contributing significantly to pharmacological activity. To enhance the clinical applicability and therapeutic efficacy of Deh, several strategies are recommended. First, the development of sustained-release formulations, nanocarrier-based delivery systems, or alternative administration routes may prolong its duration of action and improve patient compliance. Second, further investigation into its molecular targets and anti-inflammatory mechanisms is needed to broaden its potential use in inflammation-related disorders. Third, the establishment of PK/PD models could help elucidate the exposure–response relationship and guide dosage optimization. Moreover, given Deh's immunomodulatory and anti-inflammatory properties, future research could explore its therapeutic potential in chronic inflammatory diseases such as rheumatic conditions and viral infections. In conclusion, Deh holds strong promise as a candidate for natural product-based drug development, but further studies on formulation optimization and pharmacodynamic mechanisms are essential to support its clinical translation.

## Figures and Tables

**Table 1:** Basic information on experiments related to the pharmacological effects of dehydroandrograpolide and its mechanisms.

Refs	Research model	Phenotype/pathways	Dehydroandrograpolide dose (way)	Effect
Anti-inflammatory effect and mechanism				

[14]	LPS-induced ALI mice 3 mg/kg, intratracheal cell: LPS-induced RAW264.7, 100 ng/mL	NF-κB/Akt, inhibition of inflammatory cytokine production	Mice: 3, 5, 10 mg/kg, intratracheal cell: 2.5, 5, 7.5, 10, 12.5 μM	IL-6, TNF-α, NF-κB p65, Akt Ser473↓ α7nAchR↑
[15]	Cholestatic liver injury cell: kupffer cells	TLR4/NF-κB, inhibition of inflammatory cytokine production	Mice: 25 or 50 mg/kg, intraperitoneally cell: 1, 2, 4 µM	IL-6, TNF-α, ALT, TBIL, NF-κB↓
[16]	Mastitis, mammary injection LPS, cell: EpH4-Ev	AMPK/beclin/ULK1 signaling pathway	Mice: 100 mg/kg, oral gavage Cell: 1, 5, 10, 25, 50, 100, 250, 500 μM	MPO, IL6, IL-1β, TNF-α, COX2, iNos↓
[17]	LPS-induced ALI mice 2 mg/kg, intratracheal cell: LPS-induced BMDMs, 500 ng/mL	Akt/Nrf2 pathway, NLRP3-mediated pyroptosis	Mice: 12.5, 25, 50 mg/kg 10, 20, 40 μM	ROS, Akt/Nrf2, NLRP3↓
[18]	Collagen-induced arthritis (CIA) rats	Inhibited neutrophils activation via binding to LMIR3	Mice: 2 mg/kg, oral gavage	LPS/TNF-α↓, SHP-1, SHP-2↑

Antitumor effect and mechanism				

[19]	SW620 Cell	A new TMEM16A inhibitor	5 μM	TMEM16A↓
[20]	SCC9 and HSC-3 cell	Suppressed MMP expression	Mice: 20,40 mg/kg, oral gavage cell: 10, 20, 40 μM	ERK1/2, p38, JNK/2, MMP2↓ TIMP-2↑
[21]	Osteosarcoma cell	Inhibition of proliferation and cell cycle	1.25, 2.5 μM	SATB2/EMT↓
[22]	SAS and OECM-1 human oral cancer cell	Autophagy inducer	25, 50, 100 μM	JNK1/2, LC3II↑, Akt, p38, p53↓
[23]	Gastric cancer	Inhibition of tumor metastases	NA	Such as MMP9, MMP12, CTSB, ESRRG
[24]	Ishikawa cell	Inhibition of proliferation and induction of apoptosis	40, 160, 320 μmol/L	Caspasce-3↑, Bcl-2↓

Hepatoprotective effect				

[25]	Acute liver injury mice cell: human LX-2 cells	Inhibition of the activation of HSCs	Mice: 100 mg/kg, oral gavage cell: 5, 10, 20 μM	TGF-β, Col1a1, α-SMA, Mmp2, ALT↓ Mrp3↑
[26]	CCL4 induced hepatic fibrosis in mice	Reduction of oxidative stress injury, inhibition of hepatocyte apoptosis	100 mg/kg, oral gavage	Caspasce-3, bax, ALT, AST, MDA↓ Bcl-2, SOD↑
[27]	Acetaminophen-induced acute liver failure in C57BL/6 mice, 500 mg/kg	Affects autophagy, energy metabolism and antioxidant capacity	Mice: 20, 60, 100 mg/kg, oral gavage	ALT, AST, MPO, ROS, p-PI3K/PI3K↓ NRF2, p-AMPKα/AMPKα↑

Antiviral effect				

[28]	SPC-A-1 cells	Increased expression of antimicrobial peptides	80 mM	hBD-1 mRNA↑
[29]	Human lung epithelial (A549) cells Madin–Darby canine kidney (MDCK) cells165 kidney (MDCK) cells	Inhibition of H5N1 virus ribosome replication	10 μg/mL	TNF-α, IL-6, IL-8, IFN-α, IL-1 β, IFN-β↓ CXCL-10, CCL-2↓
[30]	HCT-116 intestinal cells	Increased expression of antimicrobial peptides	1, 10, 25, 50, and 100 μM	p38 MAPK, hBD-2↑
[31]	Zika virus (ZIKV) in vero cells, Huh7 cells and A549 cells	Inhibition of methyltransferase activity	0.08, 0.4, 2, 10, and 50 μM	ZIKV NS5 MTase (methyl transferase) enzymatic activity↓

Suppression of allergic reactions				

[32]	OVA-induced mice, intratracheal Cell: human mast cells	By inhibiting mast cell degranulation	Mice: 0.25, 0.5, 1 mg/kg, oral gavage Cell: 12.5, 25, 50 μM	Histamine, TNF-a, MCP-1, IL-8, IL-13, and IL-4↓
[33]	MRGPRX2-induced mice Cell: human mast cells	Deh targets CD300f and negatively regulated MRGPRX2-induced pseudo-allergic reaction	Mice: 0.5, 1, 2 mg/kg, oral gavage Cell: 25, 50, 100, 200 μM	Histamine, tryptase, TNF-α, MCP-1, and CXCL2↓

**Table 2:** Deh main pharmacokinetic parameter.

Year refs.	Subjects	Dose	Deh parameters			
			*T*_1/2_ (min)	*T*_max_ (min)	*C*_max_ (ng/mL)	AUC_0–∞_ (ng/min/mL)
2007 [53]	Human (*n* = 8)	Chuan xinlian tables, oral	3.62 ± 1.16 h	1.50 ± 0.21 h	147.30 ± 53.29 μg/mL	256.63 ± 64.18 μg h/L
2009 [54]	Chicken (*n* = 10)	5 g/kg, Oral andrographis suspension	143.00 ± 12.80	37.20 ± 2.80	1.44 ± 0.09	329.10 ± 25.50 μg/min/mL
2009 [55]	Rats (*n* = 6)	About 209 mg/kg, oral	8.8 h	48 min	5.46 μg/mL	34.65 μg h/mL
2012 [56]	Rats (*n* = 4)	120 mg/kg, oral Deh	121.66 ± 28.47	54.00 ± 13.42	4.19 ± 1.76 μg/mL	935.20 ± 491.99 μg/min/mL
2012 [56]	Rats (*n* = 4)	24 mg/kg, intravenous Deh	144.07 ± 13.03	—	—	1568.79 ± 403.40 μg/min/mL
2015 [51]	Dogs (*n* = 5)	About 7 mg, oral *A. paniculata* tablet	3.13 ± 1.19 h	1.15 ± 0.49 h	7.68 ± 2.32	27.92 ± 8.49 ng h/mL
2021 [52]	Rats (*n* = 8)	5 g/kg Xiaoyan lidan formula, oral	3.12 ± 0.61 h	1.00 ± 0.49 h	47.50 ± 8.46	232.00 ± 21.42 ng h/mL
2022 [57]	Rats (*n* = 8)	About 126 mg/kg, oral	2.28 ± 1.69 h	0.28 ± 0.09 h	892.35 ± 147.60 ng/L	2,355.73 ± 560.39 ng h/mL
2024 [58]	Mice (*n* = 5)	20 mg/kg, oral Deh	128.72 min	30 min	770.21 ± 588.28	85.47 mg/L min

## Data Availability

Data sharing is not applicable to this article as no datasets were generated or analyzed during the current study.
